# Targeting the pro-angiogenic forms of VEGF or inhibiting their expression as anti-cancer strategies

**DOI:** 10.18632/oncotarget.13942

**Published:** 2016-12-15

**Authors:** Mélanie Guyot, Caroline Hilmi, Damien Ambrosetti, Marco Merlano, Cristiana Lo Nigro, Jérôme Durivault, Renaud Grépin, Gilles Pagès

**Affiliations:** ^1^ University of Nice Sophia Antipolis, UMR CNRS 7284/U INSERM 1081, France; ^2^ Bayer S.A.S, Crop Science Division, BCS Fr-BCSF-RDFR-DVPT-TOX-TOXR, Toxicology Research, France; ^3^ Centre Hospitalier Universitaire (CHU) de Nice, Hôpital Pasteur, Central laboratory of Pathology, France; ^4^ Medical Oncology Department, S. Croce and Carle Teaching Hospital Cancer Genetics and Translational Oncology Laboratory Cuneo, Italy; ^5^ Centre Scientifique de Monaco, Monaco Principality

**Keywords:** VEGF/VEGFxxxb, angiogenesis, renal cell carcinoma, splicing, SRSF1

## Abstract

Tumor growth relies on oxygen and blood supply depending on neo-vascularization. This process is mediated by the Vascular Endothelial Growth Factor (VEGF) in many tumors. This paradigm has led to the development of specific therapeutic approaches targeting VEGF or its receptors. Despite their promising effects, these strategies have not improved overall survival of patients suffering from different cancers compared to standard therapies. We hypothesized that the existence of anti-angiogenic forms of VEGF VEGFxxxb which are still present in many tumors limit the therapeutic effects of the anti-VEGF antibodies bevacizumab/Avastin (BVZ). To test this hypothesis, we generated renal cell carcinoma cells (RCC) expressing VEGF165b. The incidence of tumors xenografts generated in nude mice and their growth were inferior to those obtained with control cells. Whereas BVZ had no effect on control tumors, it slowed-down the growth of tumor generated with VEGF165b expressing cells. A prophylactic immunization against the domain discriminating VEGF from VEGFxxxb isoforms inhibited the growth of tumor generated with two different syngenic tumor cell lines (melanoma (B16 cells) and RCC (RENCA cells)). Purified immunoglobulins from immunized mice also slowed-down tumor growth of human RCC xenografts in nude mice, producing a potent effect compared to BVZ in this model. Furthermore, down-regulating the serine-arginine-rich splicing factor 1 (SRSF1) or masking SRSF1 binding sites by 2’O-Methyl RNA resulted in the increase of the VEGFxxxb/VEGF ratio. Therefore, a vaccine approach, specific antibodies against pro-angiogenic forms of VEGF, or increasing the VEGFxxxb/VEGF ratio may represent new prophylactic or pro-active anti-cancer strategies.

## INTRODUCTION

Angiogenesis is a physiological phenomenon leading to the establishment of the vascular tree during development. Angiogenesis is a tight balance between several actors some providing angiogenesis while others tend to block it. Tumor angiogenesis is widely triggered by the Vascular Endothelial Growth Factor (VEGF) over-expression especially the main sub-family VEGF-A (named thereafter VEGF). Therefore, VEGF has been targeted in different diseases such as retinopathies and also in different cancers in association with standard chemotherapy. Bevacizumab (BVZ) a humanized neutralizing monoclonal antibody targeting VEGF has obtained FDA approval in combination with chemotherapy for colon, breast, lung and kidney cancers [1–4]. Although BVZ has proved to improve progression-free survival it still does not increase overall survival. After a decrease of the tumour size, a relapse with a particularly aggressive form of the disease has been described especially in clear renal cell carcinomas [5] and breast cancers cases [6]. These results have come as a great disappointment as BVZ was expected to be beneficial to the patients expressing VEGF because of its VEGF neutralization effect as decreasing tumor associated vasculature. Furthermore, anti-VEGF treatments in retinopathies (Ranibizumab, RNZ [7]) have to be used cautiously considering side effects, in particular high level of inflammation when injected into the eye [8] which could impair the efficacy of the treatment. Moreover, systemic neutralization of VEGF in mice leads to the death of a significant number of photoreceptors accompanied with a retinal function shrinking [9]. Because we aimed at providing good health while maintaining health expenses, treatments provided needed to be justified. The discovery of other isoforms of VEGF VEGFxxxb unveils a glimpse of understanding about the lack of BVZ/RNZ efficacy. VEGFxxxb isoforms result from an alternative splicing of exon 8a towards exon 8b. This splicing modifies the last six amino acids of the protein (CDKPRR for VEGF and SLTRKD for VEGFxxxb). In combination with other splicing events, seven pro-angiogenic and five anti-angiogenic isoforms of VEGF can be obtained. The mechanisms associated with splicing events depend on specific splicing factors in normal cells; the serine-arginine rich splicing factor 1 (SRSF1) promotes splicing towards the pro-angiogenic forms of VEGF whereas the serine-arginine rich splicing factor 6 (SRSF6) promotes splicing towards the anti-angiogenic forms of VEGF [10]. VEGFxxxb isoforms are anti-angiogenic or at least less angiogenic than the VEGF ones [11]. Both pro and anti-angiogenic forms are equally expressed in normal epithelial cells [11–12]. They have the same affinity for VEGF receptors but they trigger a different activation of these receptors. The VEGF/VEGFxxxb equilibrium in tumor cells is broken in favour of pro-angiogenic forms. Although they are down-regulated, the anti-angiogenic forms are still present in many tumors especially in renal cell carcinoma [12–13]. Furthermore, BVZ can bind both VEGF and VEGFxxxb explaining how some tumors can't benefit from the treatment when they are still expressing VEGFxxxb [14]. Thus the VEGF/VEGFxxxb ratio determines the path to *de novo* angiogenesis more significantly than the level of VEGF or VEGFxxxb [15]. BVZ decreases the density of vasculature but it promotes lymphatic vessel development [13] which gives hints about the relapse on anti-angiogenic treatments. In many cases, tumors shrink, but the selection of tumor cells with increased metastatic properties has been observed [16–17]. The identification of the mechanism leading to tumor escape may give the opportunity to personalize therapeutic approach. Patients’ specificities led us to focus on BVZ's role regarding VEGF/VEGFxxxb regulation. One of the current main goals is to adapt therapy to each patient to get the best response with minimal side effects. Whereas specific antibodies against VEGFxxxb are commercially available, antibodies specifically directed against the pro-angiogenic forms of VEGF do not exist, yet. Obviously the lack of relevant tools constitutes an obstacle to the recognition of VEGF as a pertinent prognostic factor. If intra-tumor VEGFxxxb is still present, BVZ has no effects on overall survival of patients with metastatic colon carcinoma [18]. This pivotal study favours a systematic detection of the VEGF/VEGFxxxb ratio before the administration of BVZ. Currently, only available anti-VEGF antibodies (BVZ) are recognized as having the same affinity with VEGF and VEGFxxxb [14]. We suspected that the other anti-VEGF treatments currently developed VEGF-trap/Aflibercept corresponding to parts of extra-cellular domain of VEGF receptors 1 and 2 would be confronted to the same problem [19] since this domain has the same affinity for VEGF and VEGFxxxb isoforms.

Consequently, we have been led to hypothesize that specifically targeting the pro-angiogenic forms of VEGF would have a major impact on tumor growth because the VEGFxxxb isoforms that participates in reduced tumor vascularization would not be affected. This challenging concept involves the development of antibodies directed against 6 amino-acids (CDKPRR) corresponding to the extremely conserved C-terminal domain of VEGF in mammals. Despite this short amino-acid sequence, Varey *et al*. has demonstrated the feasibility of developing specific antibodies directed against the 6 last amino-acids of the human VEGFxxxb protein (SLTRKD) [14]. The purpose of our study was to demonstrate if VEGFxxxb has the same anti-tumor role in RCC than in colon carcinoma. Repeated administrations of therapeutic monoclonal antibodies like BVZ are necessary for VEGF blockade, thereby making the treatment expensive and cumbersome. Monoclonal antibodies often show immunogenicity, thereby limiting their use. If their administration is stopped, the tumour often relapses and acquires a more invasive phenotype. Therefore we evaluated the preventive role of a prophylactic immunization against the pro-angiogenic forms of VEGF in the development of highly angiogenic tumors. Such an approach was successful in limiting tumor growth and invasiveness in models of colon cancer and melanoma [20–22] and was efficient in limiting inflammation and joint destruction in experimental arthritis [23]. In both studies, the immunized animals developed normally and wound healed efficiently indicating that anti-VEGF immunization is safe. Hence, anti-VEGF vaccination presents advantages compared to BVZ; i) it prevents the development of anti-idiotypic antibodies, ii) the immunization may be boosted by low frequency immunogen injections, iii) such immunization has a low cost. In a complementary approach, we investigated upon the relevance of targeting the SRSF1 splicing factor or its binding sites and the slowing down of the tumor growth by favoring VEGFxxxb expression.

## RESULTS

### The expression of VEGF165b has reduced the growth of RCC in nude mice and revealed a BVZ activity

**Figure 1:**
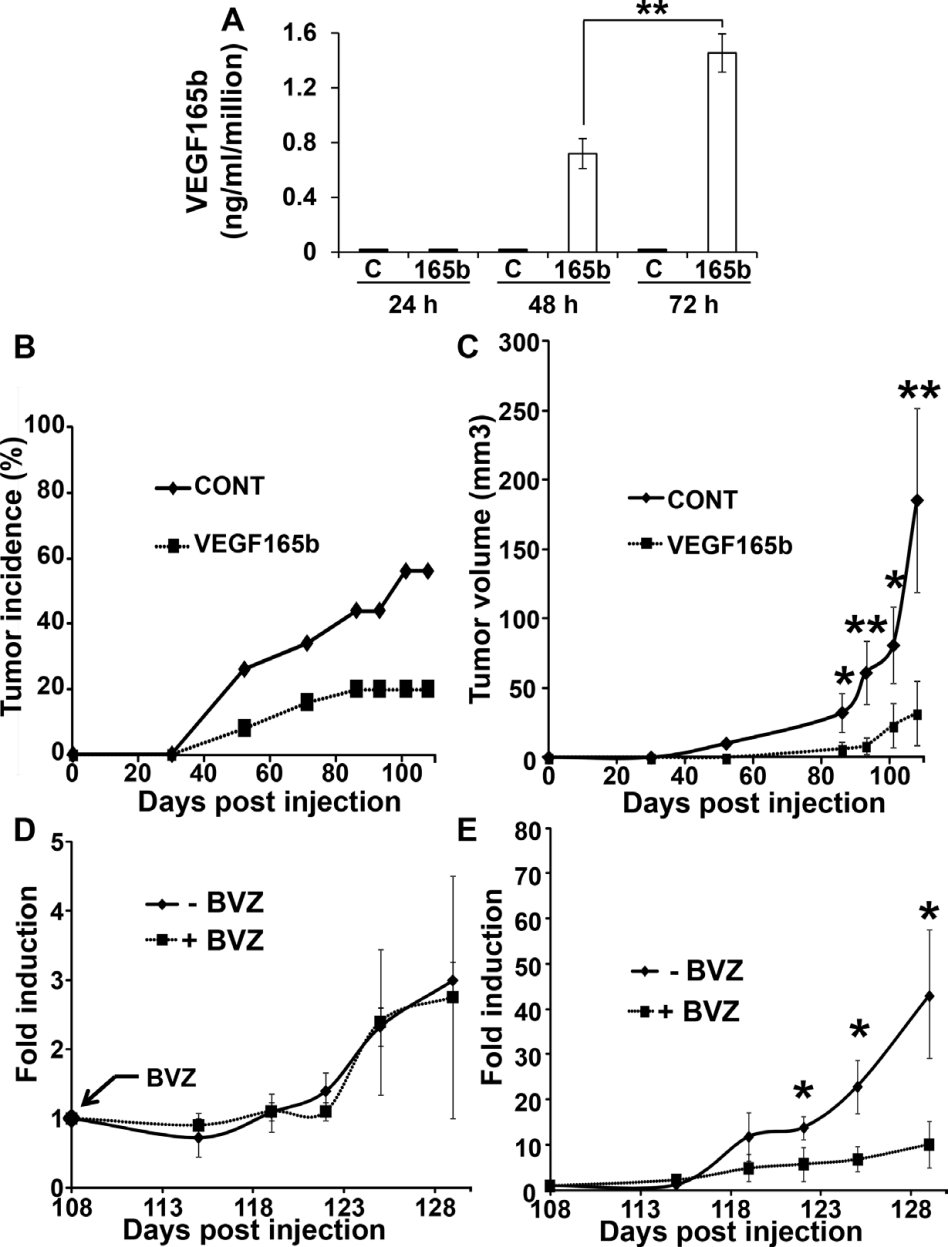
VEGF165b expression inhibits the growth of experimental RCC (**A**) 10^6^ control 786-O cells or VEGF165b-expressing 786-O cells were cultured for the indicated times. VEGF165b was quantified in cell supernatants by ELISA. The data are presented as the means ± s.d. B, C. 3.10^6^ control or VEGF165b-expressing cells were subcutaneously injected into nude mice (25 mice per group). Tumor incidence (**B**) percentage of mouse with a tumor) and tumor size (**C**) were evaluated. For the tumor size, the data are presented as the means ± s.d. Statistical differences between the size of tumors of control and treated mice are presented: *P < 0.05; **P < 0.01. (**D**, **E**) Mice described in C were treated twice a week with BVZ (150 mg per mouse +BVZ) or control IgG (- BVZ). Mice xenografted with control cells were analyzed in panel D and mice xenografted with VEGF165b-expressing cells were analyzed in panel E. The mean fold induction of tumor growth +/- standard errors (tumor volumes normalized to tumor volumes at time 108 days) was indicated for each experimental lot. Statistical significance was determined using the Student *t-test:* *P < 0.05.

We previously showed that among a cohort of 50 RCC patients, the VEGFxxxb isoforms were down-regulated in the tumor samples compared to the normal tissues [13]. However, although decreased, 70% of the tumors still expressed detectable amounts of VEGFxxxb. Hence, we hypothesized that the presence of VEGFxxxb could limit tumor growth and could serve as a predictive factor for BVZ efficacy as it is the case for colon carcinoma patients for instance [18]. For that purpose, we generated 786-O cells expressing VEGF165b (Figure 1A). The incidence of tumor formation and the tumor volume in nude mice are strongly reduced (Figure 1B, 1C). Then, we tested the influence of BVZ treatment on established tumors obtained with control or VEGF165b expressing cells 110 days after tumor cell injection. Whereas BVZ treatment had no effects on the growth of control tumors as we previously described [13] (Figure 1D), it remarkably slowed-down the growth of VEGF165b-expressing tumors (Figure 1E), a result standing out as different from those obtained on experimental colon carcinoma [14].

### Specific immunization against the pro-angiogenic forms of VEGF decreases tumor incidence and tumor growth

Considering the potent role of VEGF in tumor growth, we hypothesized that a prophylactic immunization against VEGF could limit tumor growth. Such an approach was successful in limiting tumor invasiveness in a model of colon cancer [22]. To avoid targeting the anti-angiogenic forms of VEGF, we specifically immunized mice against a tandem of the six last amino-acids of VEGF (CDKPRR-*PP*-CDKPRR) fused to the gluthation-S transferase protein. These last 6 amino acids of VEGF are highly conserved throughout the evolution process and are present in mammalian VEGF. A specific ELISA showed that most of the immunized mice contains in their plasma antibodies that specifically recognized VEGF165 and not VEGF165b. Some control mice already present a non negligible amount of anti-VEGF antibodies. According to this observation, we hypothesized that the presence of a tumor secreting high VEGF amounts may induce a self-antigen immunization process, hence, the testing of patients’ plasma with different types of cancers. Although most of plasma was not positive for anti-VEGF antibodies, at least three plasmas out of twenty were positive. We subsequently tested BVZ-treated patients’ plasma as a control of the presence of anti-VEGF antibodies. Surprisingly, some patients were negative for the presence of anti-VEGF antibodies suggesting that BVZ was degraded or cleared (Table 1).

**Table 1:** Determination of the presence of anti-VEGF antibodies in patients’ plasma samples

	Patient	OD VEGF antibody	Cancer type
**Untreated patients**	**1**	1.727	breast
	**2**	1.021	lung
	**3**	0.668	breast
	**4**	0.679	breast
	**5**	0.951	large intestine
	**6**	0.602	breast
	**7**	0.796	lung
	**8**	0.403	lung
	**9**	2.463	intestine
	**10**	0.374	large intestine
	**11**	0.690	rectum
	**12**	0.429	large intestine
	**13**	0.787	breast
	**14**	0.631	breast
	**15**	1.187	breast
	**16**	0.903	breast
	**17**	0.690	breast
	**18**	2.801	large intestine
	**19**	0.727	colon
	**20**	1.086	colon
**BVZ treated patients**	**21**	1.205	kidney
	**22**	2.672	large intestine
	**23**	2.677	breast
	**24**	3.005	rectum + lung meta
	**25**	3.146	unknown
	**26**	3.029	large intestin
	**27**	3.133	colon
	**28**	3.079	large intestine + liver meta
	**29**	3.000	large intestine + liver meta
	**30**	2.936	rectum
	**31**	0.850	kidney
	**32**	3.192	colon
	**33**	3.202	sigma
	**34**	3.204	colon
**Positive control (standard curve with BVZ)**	**BVZ 50000 ng/ml**	3.152	
	**25000 ng/ml**	3.200	
	**12500 ng/ml**	3.244	
	**6250 ng/ml**	3.266	
	**3125 ng/ml**	3.253	
	**1562 ng/ml**	3.079	
	**781 ng/ml**	2.879	
	**390 ng/ml**	2.525	
	**Blank**	0.259	

Patients with different cancer types were tested for the presence of anti-VEGF antibodies in their plasma by a sandwich ELISA. Of note patients 1, 9 and 18 were positive for anti-VEGF antibodies. As controls, patients treated with the anti-VEGF antibody bevacizumab (BVZ) were detected positive by this test except patients 21 and 31. As a positive control pure BVZ was used at different concentrations.

**Figure 2:**
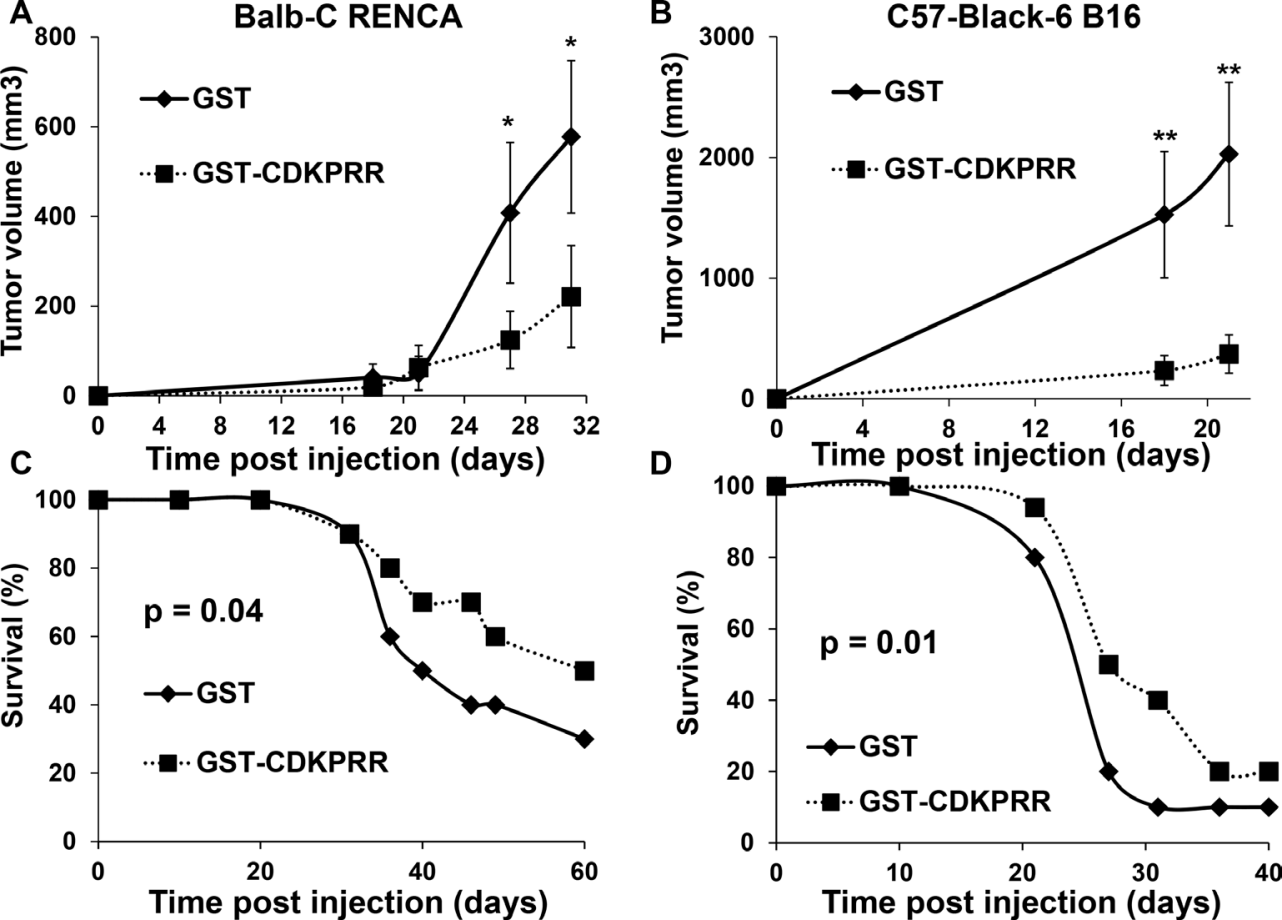
Immunization against the pro-angiogenic forms of VEGF inhibits tumor development (**A**, **C**) Balb-C mice (*n* = 20 per group) were challenged with GST or GST-CDKPRR-PP-CDKPRR every week for two months (50 mg per mouse intra-peritoneal with completed Freund adjuvant on the first week. On the second week the same protein amount was injected in the presence of incomplete Freund adjuvant. On the third week the same protein amount was injected without Freund adjuvant). For the next injection to be done, the dose of protein injected was weekly divided by half. One week after the last injection 105 RENCA cells were injected subcutaneously. Tumor size A) and survival (C) were evaluated. Statistical significance was determined: *P < 0.05; **P < 0.01. (**B**, **D**) The same protocol was applied for Black6 mice (*n* = 20 per group) and B16 cells.

To test if the presence of anti-VEGF antibodies impaired the development of experimental tumors, immunized mice were inoculated with two different syngenic models of highly aggressive renal cell carcinoma (RENCA) and melanoma (B16) cells that produced equivalent amounts of VEGF. The tumor growth was significantly decreased in anti-VEGF immunized mice (Figure 2A, 2B). The median survival time of GST CDKPRR-PPCDKPRR-immunized mice went significantly up for both model cell lines (59.1 versus 51 days *p* = 0.04 for RENCA cells and 31.8 versus 27.5 days *p* = 0.01 for B16 cells) (Figure 2C, 2D) which demonstrates the prophylactic effect of a specific immunization against the pro-angiogenic forms of VEGF.

### Purified IgG from mice immunized against the pro-angiogenic form of VEGF reduces the growth of human RCC tumors in nude mice

**Figure 3:**
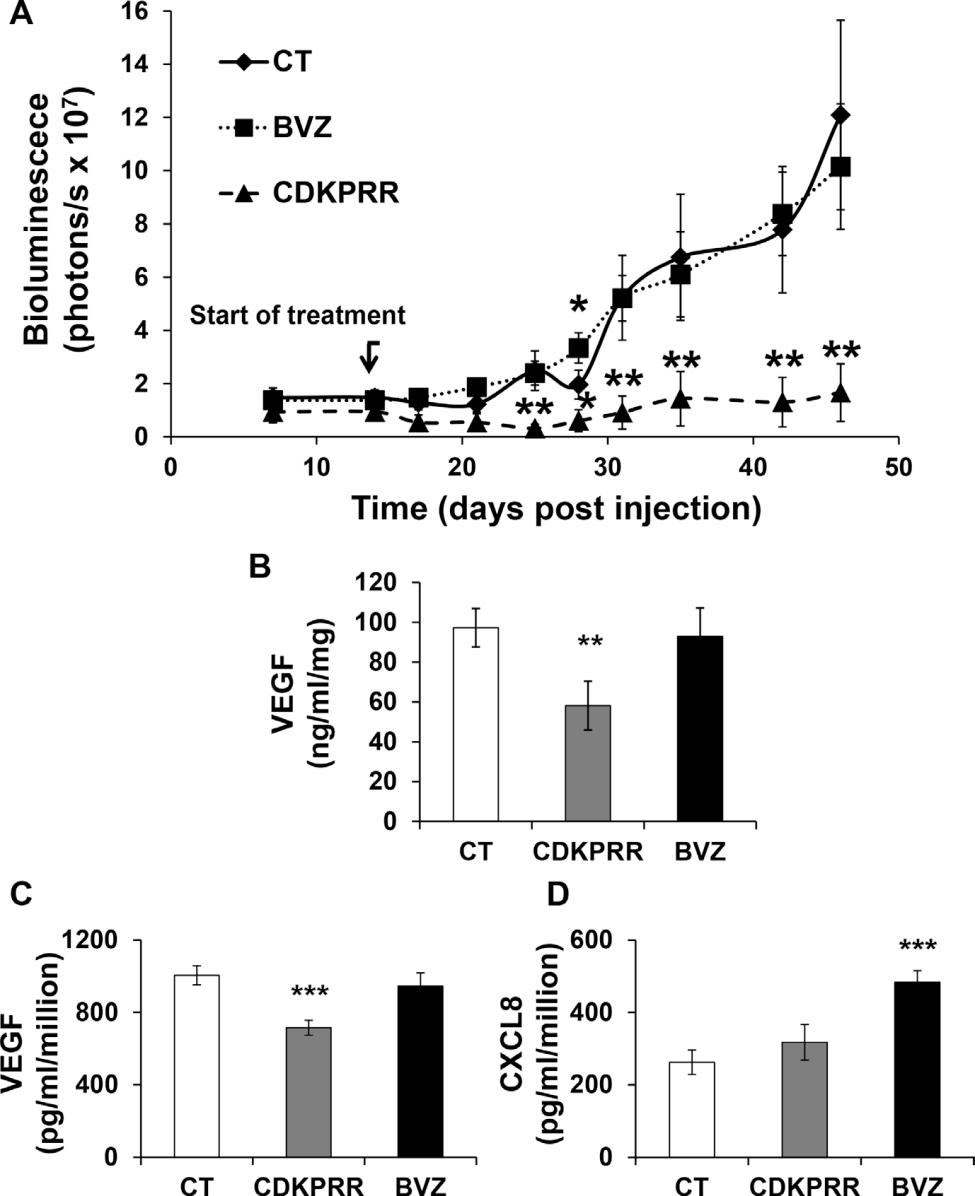
Purified IgG from mice immunized against the pro-angiogenic forms of VEGF inhibits the growth of experimental RCC (**A**) Nude mice were injected with 510^6^ 786-O cells expressing the luciferase gene. Fifteen days after cell injection, mice were treated weekly with 7.5 mg/kg of IgG purified from mice immunized with GST (CT), GST-CDKPRR-PP-CDKPRR (CDK) or BVZ. Bioluminescence was measured weekly as described previously [21]. The data are presented as the means ± s.d. Statistical differences between the size of tumors of control and treated mice are presented as follows : *P < 0.05; **P < 0.01. (**B**) Tumor extracts were tested for the presence of VEGF by ELISA. Statistically significant differences are indicated; **P < 0.01. (**C**) Cells were derived from tumors described above. Cells from four independent tumors (CT, CDKPRR or BVZ treated mice) were tested for their production of VEGF, in the supernatant, by ELISA. Statistically significant differences are indicated; ***P < 0.001. (**D**) Cells from four independent tumors (CT, CDKPRR or BVZ treated mice) were tested for their production, in the supernatant of CXCL8. Statistic differences are indicated; ***P < 0.001.

The specificity of the antibodies of control and VEGF immunized mice was first tested by ELISA. Antibodies from VEGF-immunized mice recognized human VEGF165 but not human VEGF165b. Then, they were tested for their ability to inhibit VEGF-dependent stimulation of the MAP Kinase ERK on endothelial cells. Whereas IgG isolated from control mice had no effect, IgG isolated from VEGF-immunized mice inhibited (like BVZ) the VEGF-dependent activation of ERK (Supplementary Figure S1). This experiment shows that anti-VEGF IgG inhibits cell signaling associated with proliferation events induced by VEGF on endothelial cells. The biological activity of these antibodies was further tested *in vivo* by evaluating their effect on the growth of human RCC in nude mice. Tumor growth in mice treated with IgG from GST-immunized mice or BVZ is approximately equivalent except on the thirtith day after tumor cell injection (statistically significant increase for BVZ treated mice). However, tumor growth was strongly decreased in mice treated with IgG purified from VEGF-immunized mice (Figure 3A) at a concentration equivalent to those of BVZ. Intra-tumor VEGF amounts (human plus VEGF mouse) were not modified compared to the control in the BVZ-treated tumors as we described earlier [13]. However, they were significantly decreased in the CDKPRR group (Figure 3B). The tumors from this last group of mice were too small to perform immunohistochemistry experiments. Consequently, we derived cells from independent tumors either from control IgG (CT), BVZ (BVZ) or specific anti-VEGF IgG (CDKPRR) treated mice to assess their angiogenic properties. As for tumor VEGF, VEGF165 production was equivalent in cells to CT and BVZ tumors but it was significantly reduced in CDKPRR cells (Figure 3C). We previously described that redundant pro-angiogenic factors of the ELR+CXCL cytokine family played a key role in the mechanisms of resistance to BVZ in particular CXCL8 [13]. Moreover, we have also described that another member of these family of cytokines, CXCL7 is a marker of poor prognosis for RCC [24]. The production of both cytokines was thus tested in the cells derived from the different groups of tumors [25]. CXCL7 was undetectable in cells derived from the different tumors. CXCL8 production was equivalent in CT and CDKPRR cells whereas it was increased in BVZ cells (Figure 3D). These results suggest that the treatment with antibodies inherent in the pro-angiogenic forms of VEGF prevents the *in vivo* selection of more aggressive cells with increased pro- angiogenic abilities.

### SRSF1 is a marker of poor prognosis of survival for RCC

**Figure 4:**
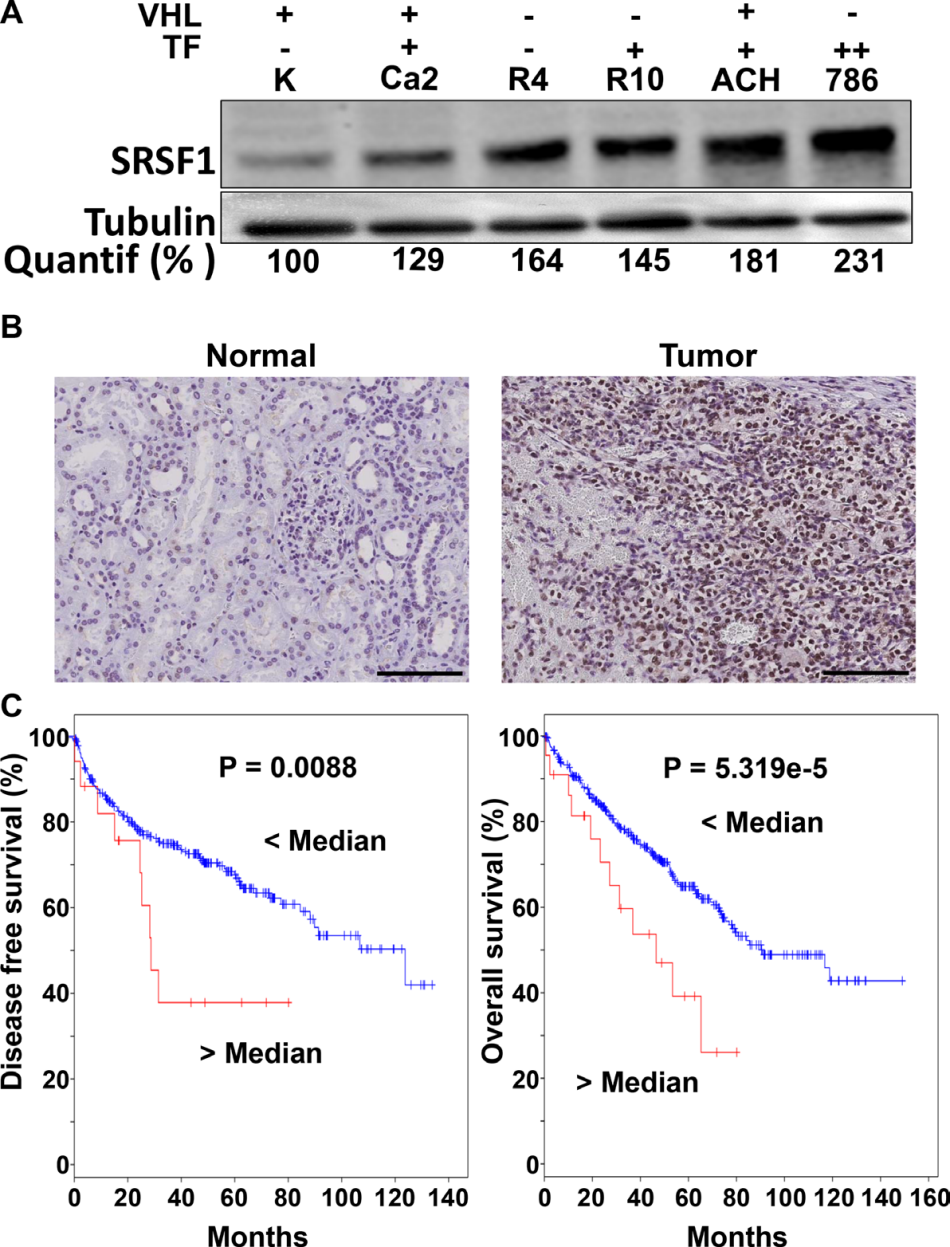
SRSF1 is overexpressed in RCC and is associated with a poor prognosis (**A**) Different RCC cell lines [Caki-2 (Ca2), (RCC4 (R4), RCC10 (R10), ACHN (ACH) and 786-O (786)] and primary normal renal cells (K) were evaluated for SRSF1 protein by immunoblot. Tubulin is shown as a loading control. Quantification of the immunoblot (Image J software) is indicated (% of normal cell expression). The pVHL status (wild-type; +; mutated; - and the ability to form tumors in nude mice (-, no tumors; +, tumors < 1 cm3 60 days after injection; ++, tumors > 1 cm3 60 days after injection) are indicated. (**B**) SRSF1 was detected by immuno-histochemistry in normal or tumor tissues (images representative of the independent normal/tumor tissues). Scale bar: 20 μm; representative image of three independent samples. (**C**) In silico analysis of the effect of SRSF1 expression levels on overall survival of RCC 534 patients and disease free survival (http://www.cbioportal.org). Kaplan–Meier curves are shown.

Nowak et al. described that the SRSF1 splicing factor favors the splicing of pre-VEGF mRNA towards expression of the pro-angiogenic form of VEGF in normal cells [10]. This result prompted us to test the role of SRSF1 as a factor of poor prognosis in RCC. SRSF1 expression was tested in different classified RCC cell lines according to their pVHL gene status and their ability to form tumors in mice [26]. We also chose as negative controls, cells isolated from the biopsies of normal kidney tissue [27]. Except for RCC4 cells that do not form tumors in mice, the ability to rapidly form tumors superior to 1 cm3 in nude mice is correlated with the highest levels of SRSF1 (Figure 4A) [13, 24]. Moreover, SRSF1 was more expressed in tumor tissues compared to their normal counterparts (Figure 4B). The analysis of online databases confirmed that increased expression of SRSF1 is correlated with shorter free disease and overall survival (Figure 4C).

### SRSF1 down-regulation slows-down tumor development

**Figure 5:**
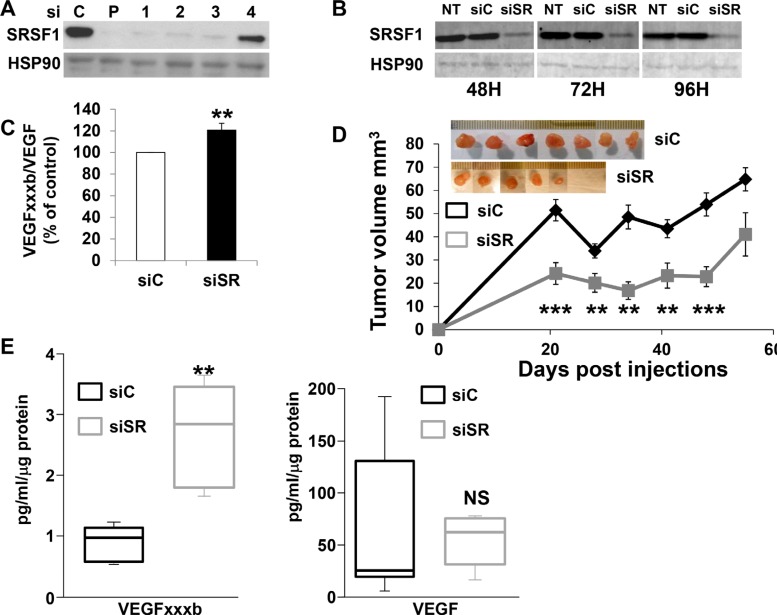
SRSF1 down-regulation delays tumor growth (**A**) SRSF1 expression was tested in 786-O cells expressing scramble (C), a mix of four independent or single siRNA against SRSF1 (P or 1–4). SRSF1 protein expression was tested by immunoblotting 48 hours after transfection. HSP90 is shown as a loading control. (**B**) Untransfected (NT) or transfected 786-O cells with scramble or siRNA directed against SRSF1 (siRNA 1, see part A of the Figure) were tested for the presence of SRSF1 48, 72 i.e. 96 hours after transfection. HSP90 is shown as a loading control. (**C**) Semi quantitative PCR was used to quantify the relative amount of VEGF165 and VEGF165b mRNA. The Figure showed the relative quantification of the VEGF165b/VEGF165 ratio of three independent experiments. **P < 0.01. (**D**) 3 × 106 si control (siC) or si SRSF1 (siSR) transfected cells were subcutaneously injected into nude mice (*n* = 10 per group). Tumor volume was measured weekly. Results are presented as the mean + SD. Statistical differences between the size of tumors of siC and siSR mice are presented: **P < 0.01, ***P < 0.001. An image of the tumors is shown. (**E**) VEGFxxxb and VEGF amounts were determined by ELISA. **P < 0.01, NS, non significant.

VEGFxxxb mRNA and protein were undetectable in 786-O cells, hence we hypothesized that SRSF1 down-regulation should decrease tumor growth by increasing the VEGFxxxb/VEGF ratio and thus by inhibiting tumor vascularization. To test this hypothesis, SRSF1 was down-regulated by siRNA. Surprisingly, SRSF1 inhibition expression persisted for a long period of time (Figure 5A, 5B). Whereas VEGF mRNA amounts were not modified by siRNA, the inhibition of SRSF1 was associated with a low but reproducible increase in VEGFxxxb mRNA (1.2 fold increase *p* = 0.0015) and subsequently in the VEGFxxxb/VEGF ratio up-regulation (Figure 5C and Supplementary Figure S2). As a matter of fact, cells down-regulated for SRSF1 (Figure 5D) were injected in nude mice to test their ability to form tumors. At the beginning of the experiment, a significant tumor volume decrease was observed among the siSRSF1 group probably reflecting a delay in blood vessel formation. However, the tumor tends to grow equally sixty days after cell injection (Figure 5D). No difference in VEGF expression was detected in tumors generated with siC and siSR transfected cells. However, VEGFxxxb was up-regulated in tumors from siSR transfected cells, which was consistent with the increased VEGFxxxb/VEGF ratio observed before injection in nude mice and with the delayed tumor growth (Figure 5E).

### SRSF1 binds the mRNA domain which differentiates VEGF pro and anti-angiogenic forms

**Figure 6:**
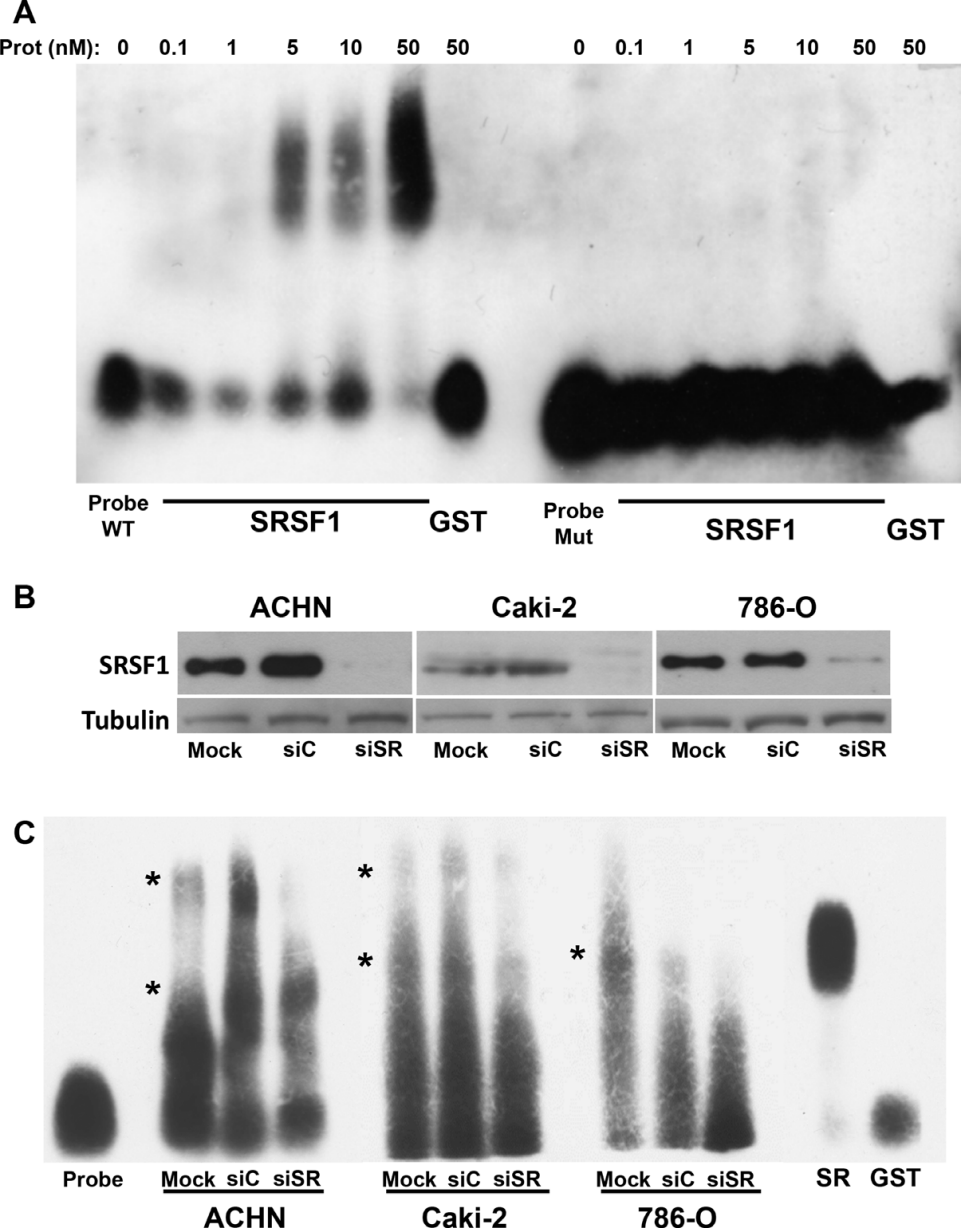
SRSF1 directly interacts with the VEGF RNA domain discriminating VEGF and VEGFxxxb isoforms (**A**) REMSAs were performed by incubating the labeled wild-type (WT) or by mutating VEGF RNA probe with increasing amount of purified GST-SRSF1 or GST in SRSF1 binding sites handling. Free probe was also shown as a control. (**B**) SRSF1 expression was tested in mock-transfected (Mock) (NT) or transfected ACHN, Caki-2 and 786-O cells with scramble or siRNA directed against SRSF1. Tubulin is shown as a loading control. (**C**) REMSAs were performed by incubating the labeled wild-type (WT) or mutating VEGF RNA probe with 1 mg of total extracts of mock in SRSF1 binding sites, si RNA (siC) or SRSF1 siRNA (siSR) transfected cells handling. Free probe alone and in the presence of 50 nM of GST or GST-SRSF1 were also shown as controls. Asterisks show the retarded bands which intensity was decreased when cells extracts from siSRSF1-transfected cells were used in comparison to the retarded bands observed with extracts from mock or siC transfected cells.

**Figure 7:**
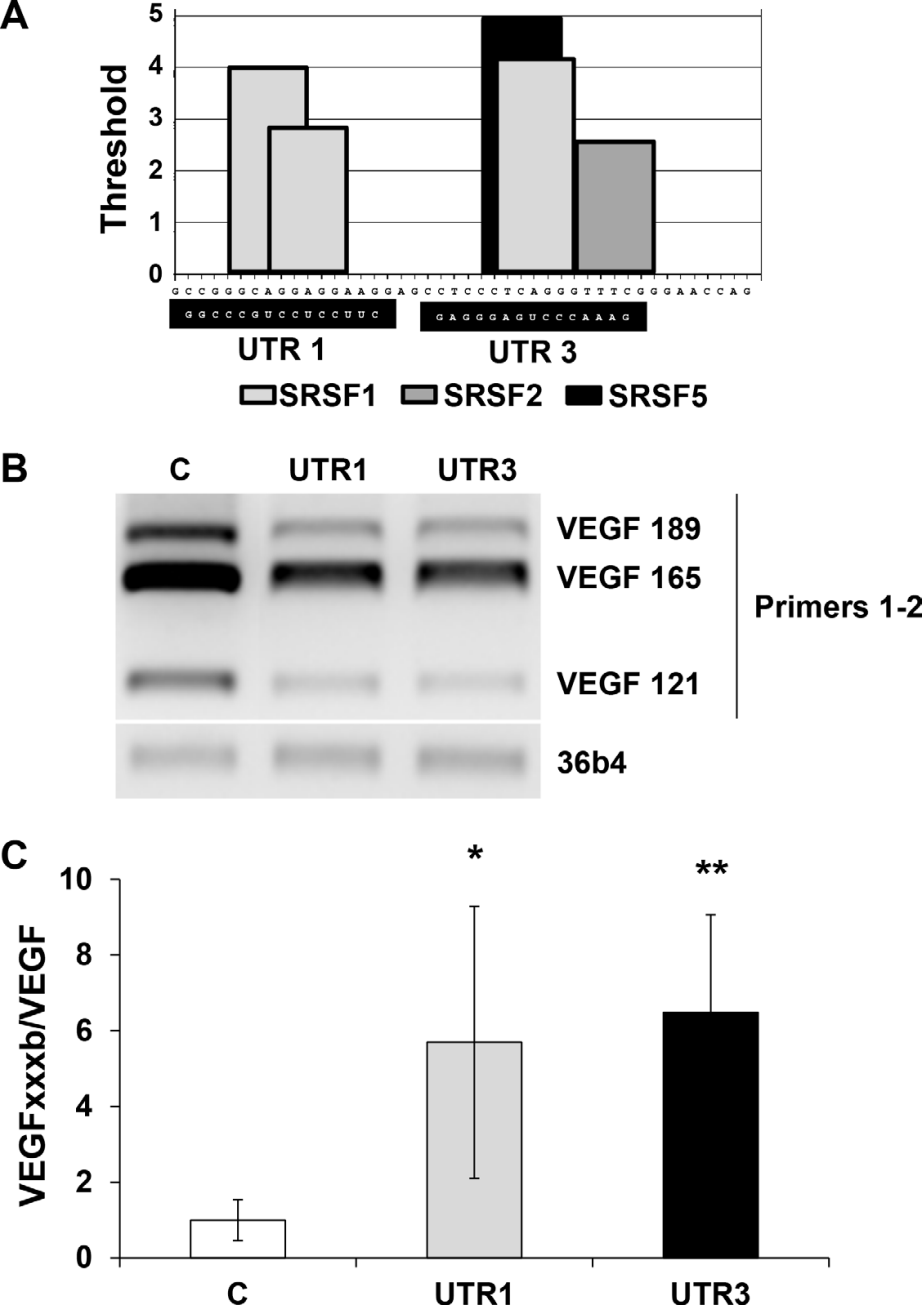
Prevention of SRSF1 binding by 2′O methyl RNA complementary of SRSF1 binding sites increased the VEGFxxxb/VEGF ratio by down-regulating VEGF expression (**A**) Schematic representation of the SRSF1 binding sites in the VEGR RNA domain differentiating VEGFxxxb and VEGF isoforms determined by in silico analysis using ESE finder. The 2′O methyl RNA sequences are shown on a black font. (**B**) Analysis of the different forms of VEGF mRNA (VEGF189, 165, 121) by analytical PCR using primers described in Supplementary Figure S2, A. 36B4 is shown as a loading control. (**C**) Analysis of VEGFxxxb and VEGF in the supernatant of scramble, UTR1 and UTR3 transfected cells by ELISA. The VEGFxxxb/VEGF ratio is shown. *P < 0.05; **P < 0.01.

To point to molecular mechanism linking the regulation of the VEGFxxxb/VEGF ratio and SRSF1, we investigated on its ability to bind to consensus sequences present in exon 8a or 8b of the VEGF pre mRNA/mRNA (Supplementary Figure S3). Nowak et al. described that SRSF1 binding sites were located in a part of VEGF that discriminates the VEGF and VEGFxxxb mRNA by using the ESE finder software. To demonstrate a real binding of SRSF1 on this domain we performed RNA shift assays using this RNA domain as a probe. Recombinant SRSF1 bound the wild-type probe in a dose-dependent manner whereas the mutation of the SRSF1 consensus sites prevented binding (Figure 6A). Cytoplasmic extracts from different RCC cells down-regulated for SRSF1 by siRNA were used in equivalent experiments (Figure 6B). SRSF1 down-regulation suppressed specific retarded bands suggesting that SRSF1 directly binds to the probe (Figure 6C). We hypothesized that the binding of SRSF1 enhances splicing towards the pro-angiogenic VEGF but also prevents the recognition of the domain involved in splicing towards the anti-angiogenic forms of VEGF by the splicing factor SRSF6 [10], hence we used 2′O-methyl RNA sequences complementary to the different zones containing SRSF1 binding sites in order to prevent SRSF1 binding from its target sequences (Figure 7A). Independent 2′O methyl sequences significantly decreased the expression of the different pro-angiogenic forms of VEGF mRNA (VEGF189, 165, 121, Figure 7B). Nested PCR using primers 3 and 4 for VEGF165 and primers 3 and 5 for VEGF165b showed that the VEGF165 and VEGF165b mRNA isoforms were down-regulated by the 2′O methyl sequences (Supplementary Figure S2A, S2B). This result suggests that inefficient splicing resulted in the degradation of pro- and anti-angiogenic VEGF mRNA isoforms. ELISA tests showed that the VEGFxxxb/VEGF ratio was increased on treatment with 2′O-methyl RNA (Figure 7C).

## DISCUSSION

We have recently shown that levels of VEGF165b are usually reduced in tumors compared to normal tissue. We have shown that in 20% of tested RCC VEGF165b levels are higher than those present in normal tissue [13]. The presence of VEGF165b in tumors may explain at least in part why some patients do not respond to BVZ or develop resistance during treatment. BVZ binds with the same affinity to pro and anti-angiogenic forms of VEGF. It inhibits at the same time the pro- and anti-angiogenic forms of VEGF which probably limits its efficacy [14]. The expression of these anti-angiogenic forms in patients could predict the failure of anti-VEGF therapies. In these tumors, high levels of VEGF expression are correlated with low survival rates [28] though VEGF has no predictive value regarding the efficacy of anti-angiogenic in RCC [29]. The presence of anti-angiogenic forms may explain this paradox. Current tools do not allow a specific detection of both forms. The development of specific antibodies against the pro-angiogenic forms is a major issue. Such antibodies would test whether the expression of pro-angiogenic forms predicts the effectiveness of anti-angiogenic treatments in patients. So solely targeting those forms of VEGF appeared as more relevant to limit angiogenesis and tumor growth. Our goal was to validate the efficacy of an antibody specific of the pro-angiogenic forms of VEGF in the treatment of RCC. For this purpose, we have created two distinct strategies among mice. The first one was a prophylactic approach which consists in immunizing mice with a peptide specific to the pro-angiogenic forms of VEGF. This immunization allows the production of antibodies inherent in these forms prior to the injection of tumor cells. This strategy has enabled us to limit the tumor incidence and to slow-down tumor growth. The second approach was a therapeutic strategy. Tumors established in mice are treated with antibodies specific to pro-angiogenic forms. This strategy allowed us to validate the benefit of these specific antibodies compared to BZV antibody reference in treating experimental RCC or other tumors for which BVZ shows a limited effect. The choice of prophylactic vaccination is based on several articles showing the effectiveness of such strategies in experimental cancer therapy in mice. An article that raised issues when published demonstrates the effectiveness of this prophylactic strategy in breast cancer treatment [30].

The target was the alpha-lactalbumin, a protein specifically expressed during lactation and over-expressed in the majority of breast cancers. Vaccination against this protein has protective and therapeutic effects in various mouse models of breast cancer. This technique is difficult to implement because the tumor antigens are often self-proteins. The use of a vaccine against these proteins can lead to autoimmune diseases. The choice of antigen is extremely important. Indeed, this one must have a very restricted expression in adults in order to minimize the problems of autoimmunity. But it must also be over-expressed in the target tumors. VEGF meets these criteria and appears to be an antigen of choice for prophylactic immunization of highly angiogenic tumors. The anti-VEGF immunization showed interesting results in the treatment of colon cancer [22], melanoma [31], ovarian cancer [32] and breast cancer [33]. The immunization method is very different from one article to another, but the results are very encouraging in all cases. These results led us to test the efficacy of this strategy for RCC. The originality of our approach was to use a specific peptide of the pro-angiogenic forms of VEGF. So we chose the specific peptide CDKPRR corresponding to the last six amino acids of VEGF as the only difference between VEGFxxx and VEGFxxxb isoforms. In the latter, this peptide is replaced by amino acids SLTRKD. Immunization with a peptide of 6 amino acids does not allow the establishment of specific antibodies. To ensure efficient production of antibodies we used the following construction for mouse immunization GST-CDKPRR-PP-CDKPRR in which the immunogen is separated by two proline residues in order to give an open and accessible structure of the protein. In previous studies, [20–22], mouse development and healing were reported as normal. In our studies, the mice were also perfectly normal even after multiple boosts. Hence, the procedure appears safe. The presence of circulating anti-VEGF antibodies in some patients as we showed in Table 1 may also suggest that it can have some physiological implications. The cost of anti-VEGF antibodies is a major concern. Our strategy may represent a good alternative to reduce the costs of the treatment for at-risk patients.

Our previous results have shown that BVZ stimulated the growth of experimental tumors in mice notably through the selection of tumor cells with increased proliferation abilities [13]. We clearly showed that purified IgG from immunized mice have a potent anti-tumor effect. However, compared to BVZ our antibodies can inhibit the effect of tumor VEGF and VEGF produced by cells of the microenvironment, as well [34]. Such an impact could explain the detrimental effects or the inefficacy of BVZ in our experimental model. Anti-VEGF has obtained FDA and EMA approval only when combined with interferon alpha for the treatment of metastatic RCC. These results also highlight the importance of interferon alpha for the efficacy of this therapeutic approach [5]. Nevertheless, the presence of anti-angiogenic forms of VEGF in a non-negligible fraction of RCC favors the use of our specific antibodies to prevent the blockade of beneficial isoforms of VEGF. Surprisingly, our results are different from those obtained by Bates et al. in colon cancers, which shows that BVZ is only efficient in tumors negative for VEGF165b [18]. In our experimental tumors over-expressing VEGF165b, BVZ inhibits tumor growth. This results may reflect the provocative results showing that VEGF165b are weakly angiogenic and not anti-angiogenic [11]. These discrepant results are in favour of the use of more specific anti-VEGF antibodies. Their humanization is the next step towards the development of new therapeutic approach for RCC. Another strategy suggested by our study will consist in forcing the expression of anti-angiogenic forms of VEGF by specifically targeting the splicing machinery. The splicing factor SRSF1 is pivotal to the expression of the proangiogenic forms of VEGF. However, SRSF1 has been shown to regulate the splicing of multiple genomic targets [35–36]. SRSF1 activity is regulated through phosphorylation events driven by the serine arginine protein kinase 1 (SRPK1). Specific inhibition of SRPK1 decreases expression of VEGFxxx isoforms and has shown therapeutic effects in experimental cancers and eye pathologies without apparent side effects [37–40]. The use of specific SRPK1 inhibitor may represent a better strategy than down-regulating SRSF1. Our experiments suggest that another technical approach used for exon jumping in experimental model of Duchenne myopathy may represent an alternative strategy [41].

As a conclusion, our study paves the way with new methods to normalize the expression of pro-angiogenic forms of VEGF and improve the current therapeutic strategies.

## MATERIALS AND METHODS

### Patients and blood samples

Thirty four blood samples of patients with different cancers were obtained at the Nice University Hospital. The presence of cancer was confirmed by histology. Patients gave their consent for the study, which was approved by our institutional review board.

### siRNA, 2′O-methyl RNA and antibodies

The following siRNA were from Dharmacon, GE Healthcare; siRNA 1; 5′-CGUGGAGUUUGUAC GGAAA-3′; siRNA 2- 5′-UGACCUAUGCAGUUC GAAA-3′; siRNA 3: 5′-UCUCGAAGCCGUAGUCG UA-3′; siRNA 4: 5′-CAGGAUUCAUGGAGCGGG A-3′. The following 2′O-methyl RNA were from Eurofins Genomics; UTR1: 5′-CUUCCUCCUGCCCGG-3′; UTR3: 5′-GAAACCCUGAGGGAG-3′. The following antibodies were used for immunoblotting and /or immunohistochemistry: anti-SRSF1 (Invitrogen); anti- α-tubulin (Fischer scientific); anti-ERK1/2 (Santa Cruz Biotechnology); anti-phospho ERK1/2 (Sigma). Cells were transfected with siRNA (0.2 μmol/L) or 2′O-methyl RNA (1 μmol/L) by using oligofectamine (Thermo Fischer Scientific).

### Molecular biology

Sense and anti-sense oligonucleotides corresponding to the CDKPRRPPCDKPRR amino-acid sequence (Sense/ 5′-ATGGATCCTGTGACAAGCCGAGGCGGCCGCCGTGTGACAAGCCGAGGCGGTGAGAATTC AT-3′; Antisense; 5′-ATGAATTCTCACCGCCTCGGCT TGTCACACGGCGGCCGCCTCGGCTTGTCACAGGA TCCAT-3′) were annealed and ligated into BamHI and EcoRI sites of the pGEX 6P1 vector (GE Healthcare, Chalfont, St. Giles, UK). The GST-CDKPRRPPCDKPRR fusion protein was produced to immunize mice. 3′. The VEGF165b cDNA was a kind gift of Dr Dave Bates [12]. It was inserted in the pLenti6/V5-DEST^®^ Gateway^®^ Vector (Life technologies, St Aubin France). Lentivirus particles were produced according to the manufacturer's recommendations and served to infect 786-O cells. Transduced cells (786-O/165b) were selected by using puromycin (5 mg/ml).

The VEGF165b sequence [12] was sub-cloned in the pLenti4/V5D-Topo vector (Invitrogen) according to the manufacturer instruction. Cells were generated with lentiviral infection and selected using puromycin (1 mg/ml). The 786-O cells used in these experiments already expressed the tetracycline repressor. This system allows the induction of the gene of interest by tetracycline for *in vitro* experiments or doxycycline for *in vivo* experiments as we described earlier [42]. For knock-down experiments, the cells were transfected with siRNA directed against SRSF1 sequence with the DharmaFECT 1 Transfection Reagent T-2001-03 (GE Health Care). Each individual or pooled siRNA were tested for their ability to block SRSF1 expression by immunoblotting. One microgram of total RNA was used for reverse transcription, using the Superscript First-Strand Synthesis System (QIAGEN, Hilden, Germany), with oligo(dT) to prime first-strand synthesis. Analytical PCR was performed by using the oligonucleotides described in Supplementary Figure S2. The oligonucleotides used for the amplification of 36B4 control mRNA are the following; forward: 5′-GCCAACCGCGAGAAGATGACCCAG-3′ and reverse 5′-CTCGAAGTCCAGGGCGACGTAGC-3′. The PCR products were analyzed by 2% agarose gels.

### Cell lines

Caki-2, RCC4, ACHN, 786-O and RENCA cells were from American Type Culture (ATCC). RCC10 cells were a kind gift from W.H. Kaelin (Dana-Farber Cancer Institute, Boston, MA) and were used in two of our published studies [24, 43]. Normal kidney cells were isolated in our laboratory and identified as kidney cells by the genetic department of our institute [27]. B16 cells were a kind gift from Dr Corinne Bertolotto (Centre Méditerranéen de Médecine Moléculaire, Nice France).

### Immunizations and treatments with anti-VEGF antibodies

Mice were given four injections 3 weeks apart of GST or GST-CDKPRR-PP-CDKPRR. For the first injection, 100 μg of protein was emulsified at a 1:1 ratio in Complete Freund›s Adjuvant (Sigma) for priming. For the second and the third injection 50 μg of protein was emulsified at a 1:1 ratio in Incomplete Freund›s Adjuvant for boosting. For the last injection, 10 μg of protein was emulsified at a 1:1 ratio in Incomplete Freund›s Adjuvant the day before injection of tumor cells or purification of IgG. IgGs were purified from hyperimmune sera by using a solid-phase protein G column as already described [22]. Tumor bearing mice were treated weekly with 7.5 mg/kg of IgG purified from mice immunized with GST (CT), GST-CDKPRR-PP-CDKPRR (CDK) or BVZ (ip injection).”

### Tumor xenograft formation and size evaluation

786-O, 786-O/165b, RENCA and B16 cells were injected subcutaneously into the flanks of 5-week-old nude (nu/nu), Balb-C (RENCA) and C57-Black-6 (B16) female mice (Janvier, France). Tumor volume (*v* = L × l2 × 0.52 [44]) was determined in parallel using a caliper.

### Measurement of cytokines

Frozen tumor tissues were lysed in cell extraction buffer (Biosource, Belgium) by using the Bertin homogenizer Precellys^®^ (Bertin Instruments). Human and mouse VEGF were measured using PeproTech ELISA kits according to the manufacturer's recommendations (PeproTech, Neuilly-sur-Seine, France). VEGF165b was measured using the Human DuoSet ELISA kit (R&D Systems, Minneapolis, USA).

### Immunohistochemistry

Samples were collected with the approval of the Local Ethics committee. Sections from blocks of formol-fixed and paraffin-embedded tissue were examined for immunostaining for SRSF1. After deparaffinization, hydration and heat-induced antigen retrieval, the tissue sections were incubated for 20 minutes at room temperature with anti-SRSF1 antibodies diluted at 1:100. Biotinylated secondary antibody (DAKO) was applied and binding was detected with the substrate diaminobenzidine against a hematoxylin counterstain.

### RNA electromobility shift assays (REMSAs)

For REMSA experiments, 30 pmol of biotinylated RNA using Biotin RNA Labeling Mix (Pierce Chemical) was combined with increasing concentrations (0, 0.1, 1, 5, 10, 50 nmol/L) of GST fusion proteins or 1 mg of cell extracts, in a previously described binding buffer [45]. The following probes were used: wild-type probe: 5′-GCCGGGCAGGAGGAAGG AGCCTCCCTCAGGGTTTCGGGAACCAG-3′; mutated probe for SRSF1 sites: 5′-GCCGGGAAGGTGGAA GGAGCCTCCTTGACGGTGTCGGGAACCAG-3′. The reaction mixture was incubated for 30 min at 30°C and treated for 15 min at room temperature with 100 U of ribonuclease T1 (Roche). When specific or nonspecific competitors were used, they were incubated for 15 min at 30°C with the proteins in binding buffer before the addition of the biotinylated transcripts. The reaction mixtures were resolved on 5% native polyacrylamide gels in 0.5 × Tris borate-EDTA (TBE) buffer. Gels were transferred to nylon N^+^ membranes in 0.5 × TBE at 400 mA and 4°C for 1 h. The RNAs were cross-linked to the membranes and detected by the lightshift electrophoretic mobility shift assay kit (Pierce Chemical) by using streptavidin-horseradish peroxidase binding and chemiluminescent detection.

### Analysis of cbioportal databases

Disease-free (DFS) and overall (OS) survival were calculated from patient subgroups with RCC (TCGA provisional) with mRNA levels of TERF2 that were 1.5 fold greater (log2) than the median value (mRNA Expression z-Scores RNA Seq V2 RSEM)). RCC tumor samples with mRNA data were selected in cbioportal (534 samples out of 538; 434 samples were analyzed for DFS and 532 for OS).

### Statistical analysis

Statistical analyses were two-sided and were performed using R-2.12.2 by Windows. Statistical comparisons were performed using the Chi-2 test or Fisher exact test for qualitative data, the Student *t-test* or Wilcoxon test for quantitative data and the Log-Rank test for censored data.

## SUPPLEMENTARY MATERIALS FIGURES


