# Supplementary material for: Targeting the pro-angiogenic forms of VEGF or inhibiting their expression as anti-cancer strategies
Source: Oncotarget. 2016 Dec 15;8(6):9174–88. doi: 10.18632/oncotarget.13942 (PMC5354723; doi:10.18632/oncotarget.13942)
Supplement: Supplementary file 1 [file oncotarget-08-9174-s001.pdf]

Targeting the pro-angiogenic forms of VEGF or inhibiting their expression as anti-cancer strategies

Supplementary Materials

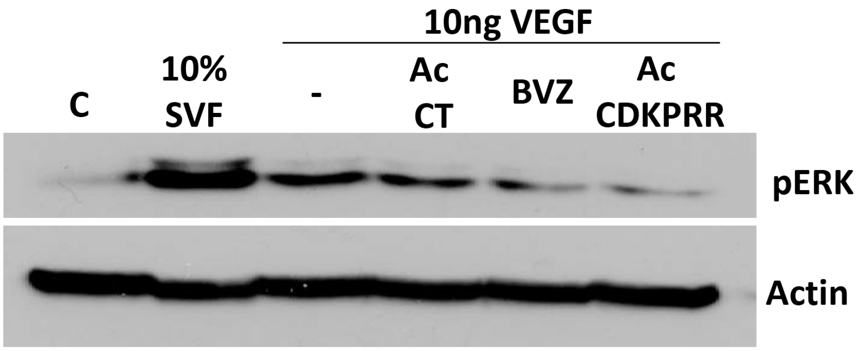

Supplementary Figure S1: Antibodies purified from immunized mice inhibit VEGF-dependent stimulation of ERK in endothelial cells. Serum starved HMVEC cells were stimulated for 10 minutes with 10 ng/ml of VEGF in the absence or presence of IgG from GST-(CT), GST-CDKPRRPPCDKPRR (CDK) or BVZ (10 µg/ml). The active phosphorylated forms of ERK were detected by immunoblotting. Actin serves as a loading control. This experiment is representative of three independent ones.

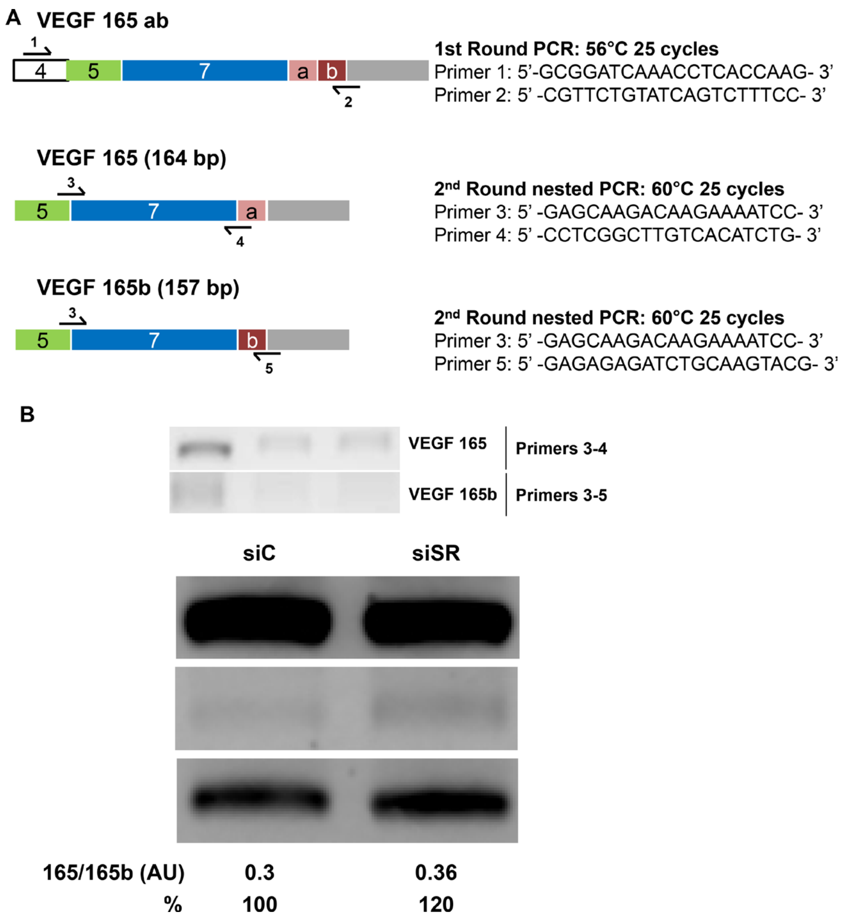

Supplementary Figure S2: Schematic representation and sequences of the primers chosen for the specific amplification of VEGF and VEGFxxxb isoforms. For the first round PCR we used primers corresponding to sequence of exon 4 (primer 1) and exon 8 (primer 2). These primers can lead to amplification of both VEGF and VEGFxxxb isoforms. For nested PCR we used a primer located between exon 5 and 7 (primer 3) and a primer located between exon 7 and 8a (primer 4, specific amplification of VEGF isoforms) or a primer located a region corresponding to the 3'UTR of VEGF mRNA (a domain common for VEGF and VEGFxxxb isoforms) and exon 8b (primer 5, specific amplification of VEGFxxxb isoforms). The sequence of each primer is shown.

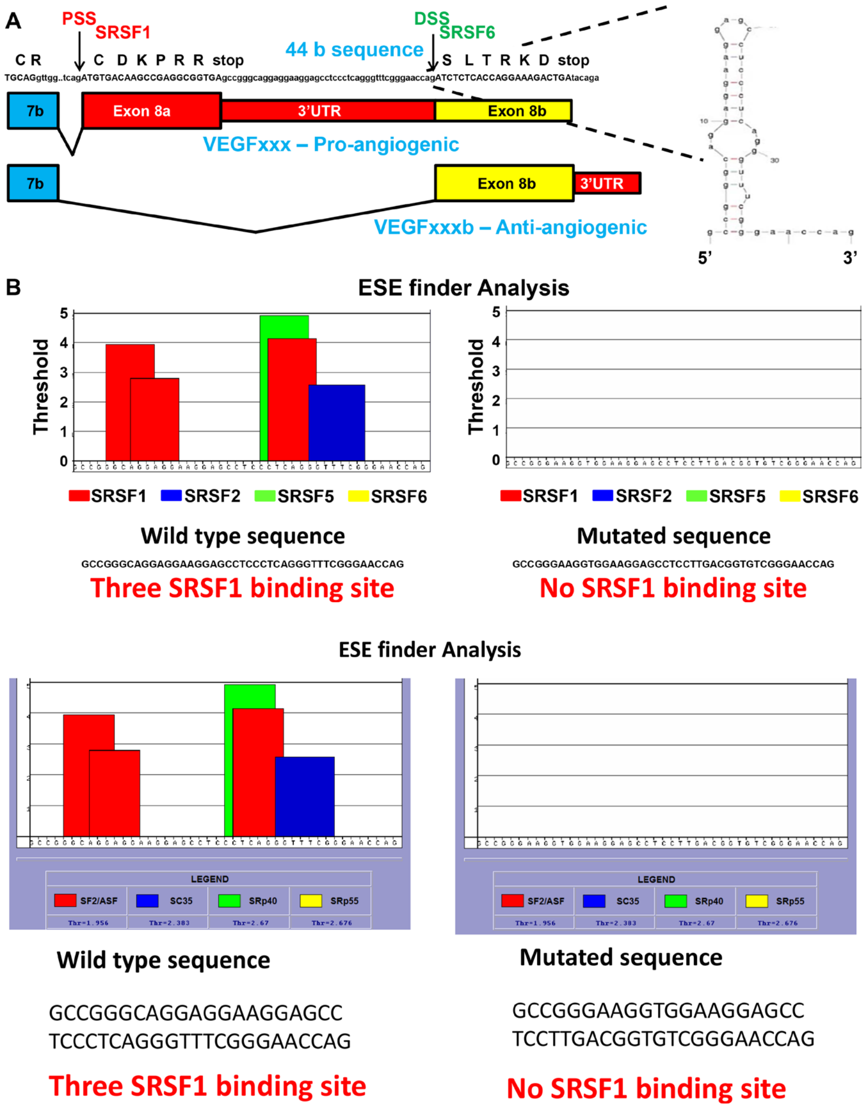

Supplementary Figure S3: Schematic representation of the domains corresponding to VEGF and VEGFxxxb isoforms. (**A**) The different exons that are involved in the generation of VEGF and VEGFxxxb isoforms are mentioned (7b, 8a 8b). The domain that differentiated VEGF and VEGFxxxb can form an hairpin structure modelized with the mFold software (http://unafold.rna.albany.edu). (**B**) The ESE finder software detected SRSF1, SRSF2 and SRSF5 binding sites within the 44 bases discriminating VEGF and VEGFxxxb isoforms. No SRSF1 binding sites were detected in the mutated sequence used in the experiments described in Figure 6.
